# Prolonged-Release Once-Daily Formulation of Tacrolimus Versus Standard-of-Care Tacrolimus in *de novo* Kidney Transplant Patients Across Europe

**DOI:** 10.3389/ti.2021.10225

**Published:** 2022-03-21

**Authors:** Klemens Budde, Lionel Rostaing, Umberto Maggiore, Giovanni Piotti, Daniela Surace, Silvia Geraci, Claudio Procaccianti, Gabriele Nicolini, Oliver Witzke, Nassim Kamar, Laetitia Albano, Matthias Büchler, Julio Pascual, Alex Gutiérrez-Dalmau, Dirk Kuypers, Thomas Wekerle, Maciej Głyda, Mario Carmellini, Giuseppe Tisone, Karsten Midtvedt, Lars Wennberg, Josep M. Grinyó

**Affiliations:** ^1^ Department of Nephrology, Charité Universitätsmedizin Berlin, Berlin, Germany; ^2^ Service de Néphrologie, Dialyse, Aphérèses et Transplantation, CHU Grenoble Alpes, Grenoble, France; ^3^ Department of Medicine and Surgery, University of Parma, Parma, Italy; ^4^ Chiesi Farmaceutici S.p.A., Parma, Italy; ^5^ Department of Infectious Diseases, West German Centre of Infectious Diseases, Universitätsmedizin Essen, University of Duisburg-Essen, Essen, Germany; ^6^ Departments of Nephrology and Organ Transplantation, CHU Rangueil, INSERM U1043, IFR–BMT, Université Paul Sabatier, Toulouse, France; ^7^ Unité de Transplantation Rénale, Hôpital Pasteur 2, CHU Nice, Nice, France; ^8^ Service de Néphrologie et Transplantation Rénale, CHRU de Tours, Tours, France; ^9^ Department of Nephrology, Hospital del Mar, Barcelona, Spain; ^10^ Department of Nephrology, Hospital Universitario Miguel Servet, IIS Aragón, Zaragoza, Spain; ^11^ Department of Nephrology and Renal Transplantation, University Hospitals Leuven, Leuven, Belgium; ^12^ Department of Surgery, Medical University of Vienna, Vienna, Austria; ^13^ Department of Transplantology, Surgery and Urology, District Hospital, Poznan, Poland, and Nicolaus Copernicus University Collegium Medicum, Bydgoszcz, Poland; ^14^ Department of Surgery and Bioengineering, University of Siena, Siena, Italy; ^15^ Transplant Unit, Tor Vergata University, Rome, Italy; ^16^ Department of Transplant Medicine, Oslo University Hospital Rikshospitalet, Oslo, Norway; ^17^ Department of Transplantation Surgery, Karolinska University Hospital, Stockholm, Sweden; ^18^ Department of Nephrology, Hospital Universitari de Bellvitge, University of Barcelona, Barcelona, Spain

**Keywords:** kidney, transplantation, immunosuppression, tacrolimus, pharmacokinetics, LCPT

## Abstract

**Background:** Tacrolimus is the calcineurin inhibitor of choice for preventing acute rejection episodes in kidney transplant patients. However, tacrolimus has a narrow therapeutic range that requires regular monitoring of blood concentrations to minimize toxicity. A new once-daily tacrolimus formulation, LCP-tacrolimus (LCPT), has been developed, which uses MeltDose™ drug-delivery technology to control drug release and enhance overall bioavailability. Our study compared dosing of LCPT with current standard-of-care tacrolimus [immediate-release tacrolimus (IR-Tac) or prolonged-release tacrolimus (PR-Tac)] during the 6 months following *de novo* kidney transplantation. Comparisons of graft function, clinical outcomes, safety, and tolerability for LCPT versus IR-Tac/PR-Tac were also performed.

**Methods:** Standard immunological risk patients with end-stage renal disease who had received a *de novo* kidney transplant were randomized (1:1) to LCPT (N = 200) or IR-Tac/PR-Tac (N = 201).

**Results:** Least squares (LS) mean tacrolimus total daily dose from Week 3 to Month 6 was significantly lower for LCPT than for IR-Tac/PR-Tac. Although LS mean tacrolimus trough levels were significantly higher for LCPT than IR-Tac/PR-Tac, tacrolimus trough levels remained within the standard reference range for most patients. There were no differences between the groups in treatment failure measures or safety profile.

**Conclusion:** LCPT can achieve similar clinical outcomes to other tacrolimus formulations, with a lower daily dose.

**Clinical Trial Registration:** https://clinicaltrials.gov/, identifier NCT02432833.

## Graphical Abstract

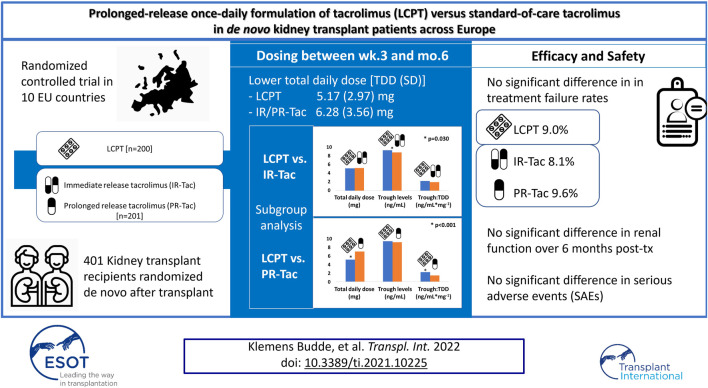

## Introduction

Tacrolimus is the calcineurin inhibitor of choice in the prevention of acute rejection episodes in kidney transplant patients (1). It has a primary role in immunosuppressive regimens and is associated with improved outcomes owing to its efficacy and beneficial effect on renal allograft function (2). There may, however, be complexities with respect to regimen optimization due to the variability of tacrolimus exposure, which is partly a function of its low bioavailability (3, 4). In addition, tacrolimus has a narrow therapeutic range that imposes regular monitoring of blood drug concentrations to maintain therapeutic target levels and minimize toxicity (5, 6). Exposure below the minimum therapeutic level puts patients at risk of graft rejection and graft failure (and indeed, recent trends for tacrolimus minimization are still producing unsatisfying results) (7), whilst overexposure is associated with increased toxicity, including development of delayed graft function and post-transplant diabetes mellitus (8).

Two formulations of tacrolimus have been available for some time: an immediate-release formulation (IR-Tac), which is dosed twice daily (3), and a prolonged-release formulation (PR-Tac), which is dosed once daily (4). These formulations exhibit considerable inter- and intra-patient variability in absorption and metabolism, affected by multiple factors including the patient’s *CYP3A5* phenotype, sex, age, concomitant medication, and diet (9–11). Therapeutic drug level monitoring is therefore mandatory, and trough levels are concentration-controlled in clinical practice (as they correlate with systemic exposure as indicated by the area under the blood drug concentration–time curve). The benefits of once-daily administration of PR-Tac must be balanced against delayed achievement of, or change in, therapeutic trough levels and the higher dose needed to achieve similar trough levels to IR-Tac (12).

A new once-daily formulation of tacrolimus is now available [Envarsus^®^, LCP-tacrolimus (LCPT)] (13). LCPT was developed using MeltDose™ drug-delivery technology in order to enhance overall bioavailability (14). This technology controls the release of the drug mainly through a more distal distribution of tacrolimus within the gut, with the potential of being less affected by first-pass metabolism due to CYP3A activity along the proximal gut wall (15, 16). Compared with IR-Tac and PR-Tac, LCPT has higher bioavailability and a flatter time concentration curve in stable and *de novo* kidney transplant recipients (17, 18), even at very low trough levels (19). LCPT demonstrated non-inferiority in clinical outcomes and similar safety profiles to twice-daily tacrolimus in both *de novo* and stable kidney transplant patients (14, 20, 21).

The present study compared LCPT with current standard-of-care tacrolimus (IR-Tac or PR-Tac according to local clinical practice) during the 6 months following *de novo* kidney transplant in a series of European centers. Because dosing may affect drug exposure, in turn impacting graft function and drug side effects, the primary objective was to compare dosing of LCPT with standard-of-care tacrolimus. Clinical outcomes, safety, and tolerability were also evaluated.

## Methods and Materials

This was a Phase IV, randomized, open-label, parallel group study, conducted in 10 European countries. The study was conducted according to the current International Council for Harmonisation Good Clinical Practice guidelines, any local guidelines, and the Declaration of Helsinki, and the study protocol was approved by Independent Ethics Committees in accordance with local requirements. All patients provided written informed consent. The study was sponsored by Chiesi Farmaceutici (NCT02432833).

### Study Population

Adults (≥18 years of age) with end-stage renal disease who received a *de novo* kidney transplant from a living or deceased donor were eligible. Patients with a known contraindication for tacrolimus or other macrolides were excluded. Key exclusion criteria included receipt of any other transplanted organ; receipt of a previous kidney transplant or of a kidney from a donor following cardiac death; receipt of a kidney with cold ischemia time of ≥30 h; receipt of a kidney from positive cross-match or ABO-incompatible donor; and current anti-human leukocyte antigen panel reactive antibody levels of >30%.

### Design and Study Drugs

Fifteen study visits were scheduled over the 6-months study period: screening [0–28 days before transplantation if possible (e.g., in the case of a living donor)]; Day 0 (kidney transplantation); Day 1 (first administration of study drug); and Days 3, 5, 7, 10, 14, 21, 28, 60, 90, 120, 150, and 180. Baseline assessments were performed at the screening visit. If this was not possible (e.g., in the case of a deceased donor), they were performed on the day of transplantation.

Patients were randomized (1:1) to receive either LCPT or standard-of-care tacrolimus according to local practice, i.e., IR-Tac (Prograf^®^; Astellas Ireland Co., Ltd., Killorglin, Ireland) or PR-Tac (Advagraf^®^; Astellas Ireland Co., Ltd., Killorglin, Ireland). A balanced, blocked, randomization scheme, stratified by study site, was prepared by the study sponsor using a computerized system. Randomization was performed using an interactive web response system after baseline assessments were complete. Randomization took place preferably after transplantation, although it was allowed before transplantation once it was certain the patient would receive the kidney. At latest, randomization took place on the day following transplantation prior to the first administration of study drug.

In accordance with the prescription insert, the starting doses of study drug were 0.17 mg/kg/day once daily in the morning for LCPT, 0.20 mg/kg/day in two divided doses (morning and evening) for IR-Tac, and 0.20 mg/kg/day once daily in the morning for PR-Tac. The first dose was administered within 24 h after surgery. All study drugs were given orally. Doses were adjusted to maintain tacrolimus whole blood trough levels within the standard reference range, i.e., 5–15 ng/ml during the first 3 months following transplantation and 5–10 ng/ml thereafter.

Permitted concomitant immunosuppressive drugs included basiliximab, mycophenolate mofetil, and corticosteroids; treatment for acute rejection included corticosteroids, T-cell and B-cell depleting antibodies, plasma exchange, and intravenous immunoglobulin.

### Endpoints and Assessments

The primary endpoint was the tacrolimus total daily dose (TDD) from Week 3 to Month 6. Secondary dosage endpoints over the whole study period were 1) tacrolimus TDD overall, by visit and by period (weekly during the first month, 1–3 months, and 3–6 months); 2) TDD normalized for weight; 3) tacrolimus trough levels overall, by visit, and by period; 4) number of times the trough level was within the standard reference range; 5) ratio of trough level to TDD (trough:TDD) overall, by visit, and by period; 6) number of dose adjustments. Pre-specified exploratory dosage endpoints included separate comparisons of LCPT with each of the other Tac formulations, (LCPT vs. IR-Tac and LCPT vs. PR-Tac) for TDD from Week 3 to Month 6, trough levels, and trough:TDD over the same period.

Secondary clinical endpoints were 1) treatment failure (composite endpoint comprising death, graft failure, biopsy-proven acute rejection, and loss to follow-up); 2) treatment discontinuation; 3) delayed graft function (defined as dialysis in the first week); 4) local diagnosis of acute rejection requiring treatment (classified as acute by the investigator and requiring additional immunosuppressive medications); 5) concomitant immunosuppressive medications. Safety assessments included adverse events (AEs), clinical laboratory tests (including for cytomegalovirus and urinary tract infections), 12-lead electrocardiogram (ECG), and vital signs.

### Data Analysis

All efficacy endpoints were analyzed in the modified intent-to-treat (mITT) population (all randomized patients who received at least one dose of study treatment and had at least one available evaluation of efficacy after baseline). The safety population included all randomized patients who received at least one dose of study drug.

All statistical tests were carried out using 2-sided 0.05 significance levels. Differences between the treatment groups were estimated with the associated 2-sided 95% confidence intervals (CI). The exact Clopper-Pearson method was used to produce the 95% CI for individual proportions (rates) that corresponded to treatment groups. Fisher’s exact test was used to compare the proportions between treatment groups. The difference in proportions between treatment groups was estimated and the associated 95% CIs provided were based on the Newcombe-Wilson method.

The primary endpoint was the average tacrolimus TDD from Week 3 to Month 6 and was compared between the two groups by applying an analysis of variance (ANOVA) model with treatment group and country as fixed effects. The adjusted least squares (LS) means in each treatment group and the adjusted LS mean difference between treatment groups were calculated with the corresponding 2-sided 95% CIs. The overall TDD (average over the whole treatment period) was analyzed in the same way.

For specific endpoints collected at several timepoints, a mixed model for repeated measures (MMRM) was performed. The model includes treatment arm, country, period, and a term for the interaction between treatment and period. Where specified, the baseline value was added as a covariate. The adjusted means in each treatment group and the adjusted mean difference between treatment groups were displayed with the corresponding 2-sided 95% CIs.

A sample size of 180 patients per study arm was planned to achieve a power of 80% to demonstrate a difference between LCPT and IR-Tac/PR-Tac of approximately −14% at a 2-sided significance level of 0.05, assuming an average TDD of 6.3 mg [standard deviation (SD) 3.0 mg] in both study arms. Assuming screening failure and discontinuation rates of 10%, 445 patients needed to be enrolled to achieve 400 patients randomized and 360 patients completing the study.

## Results

### Patient Characteristics

**FIGURE 1:**
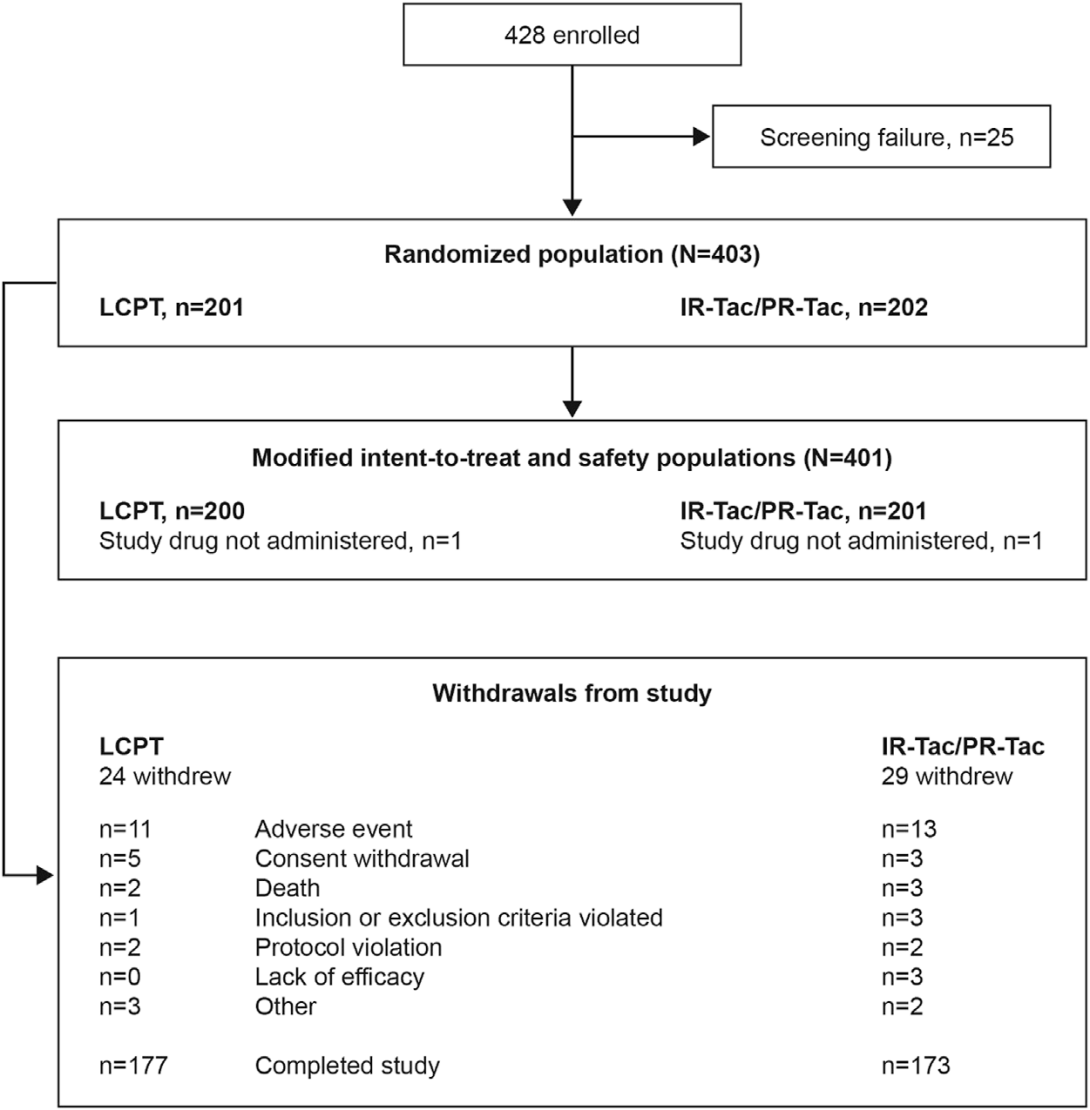
Patient disposition. IR-Tac, immediate release tacrolimus; LCPT, LCP tacrolimus; PR-Tac, prolonged release tacrolimus.

A total of 401 patients were included in the mITT and safety populations: 200 in the LCPT group and 201 in the IR-Tac/PR-Tac group (IR-Tac: 86; PR-Tac: 115), and 350 (86.8%) patients completed the study (Figure 1). Demographic and patient characteristics were similar in the LCPT and IR-Tac/PR-Tac groups; most patients were white men and the mean age was 54.3 years (Table 1).

**TABLE 1:** Baseline demographic and transplant characteristics (mITT population).

Characteristic	LCPT (N = 200)	IR-Tac/PR-Tac (N = 201)	IR-Tac (N = 86)	PR-Tac (N = 115)
Age				
Mean (SD), years	53.8 (14.2)	54.8 (14.2)	53.4 (15.1)	55.8 (13.4)
<65 years, n (%)	147 (73.5)	147 (73.1)	63 (73.3)	84 (73.0)
Male sex, n (%)	146 (73.0)	136 (67.7)	59 (68.6)	77 (67.0)
Race, n (%)				
White	195 (97.5)	192 (95.5)	84 (97.7)	108 (93.9)
Asian	2 (1.0)	3 (1.5)	1 (1.2)	2 (1.7)
Black	1 (0.5)	1 (0.5)	0	1 (0.9)
Other	2 (1.0)	5 (2.5)	1 (1.2)	4 (3.5)
Body mass index, mean (SD), kg/m^2^	26.8 (4.6)	26.0 (4.6)	25.6 (4.6)	26.4 (4.6)
Weight, mean (SD), kg	78.33 (15.07)	75.51 (14.64)	75.2 (16.2)	75.8 (13.4)
Diabetes pre-transplantation, n (%)	37 (18.5)	42 (20.9)	15 (17.4)	27 (23.5)
Time from transplant to first dose, mean (SD), hours	18.3 (8.2)	17.7 (7.5)	17.0 (8.7)	18.2 (6.5)
Pre-emptive transplantation, n (%)				
Yes	28 (14.0)	31 (15.4)	15 (17.4)	16 (13.9)
No	172 (86.0)	170 (84.6)	71 (82.6)	99 (86.1)
Type of dialysis, n (%)^a^ ^,^ ^b^				
Hemodialysis	138 (80.2)	139 (81.8)	66 (93.0)	73 (73.7)
Peritoneal dialysis	33 (19.2)	29 (17.1)	5 (7.0)	24 (24.2)
Missing	1 (0.6)	2 (1.2)	0	2 (2.0)
Time from first dialysis to transplant, median (range), months	29.3 (0, 152)	26.7 (0, 166)	32.9 (29.0)	38.7 (28.9)
Donor type				
Living	40 (20.0)	38 (18.9)	18 (20.9)	20 (17.4)
Deceased	160 (80.0)	163 (81.1)	68 (79.1)	95 (82.6)
HLA-A mismatch, n (%)				
0	31 (15.5)	33 (16.4)	18 (20.9)	15 (13.0)
1	98 (49.0)	94 (46.8)	47 (54.7)	47 (40.9)
2	66 (33.0)	68 (33.8)	20 (23.3)	48 (41.7)
HLA-B mismatch, n (%)				
0	21 (10.5)	24 (11.9)	15 (17.4)	9 (7.8)
1	88 (44.0)	99 (49.3)	41 (47.7)	58 (50.4)
2	86 (43.0)	72 (35.8)	29 (33.7)	43 (37.4)
HLA-DR mismatch, n (%)				
0	37 (18.5)	50 (24.9)	26 (30.2)	24 (20.9)
1	126 (63.0)	99 (49.3)	40 (46.5)	59 (51.3)
2	32 (16.0)	46 (22.9)	19 (22.1)	27 (23.5)
Maximum PRA, n (%)				
0%	171 (85.5)	182 (90.5)	73 (84.9)	109 (94.8)
≥1%	19 (9.5)	8 (4.0)	7 (8.1)	1 (0.9)

a Percentage was based on the number of subjects with pre-emptive transplantation answered as “no”.

b Type of dialysis has been derived for subjects with pre-emptive transplantation answered as “no”

HLA, human leukocyte antigen; IR-Tac, immediate release tacrolimus; LCPT, LCP tacrolimus; mITT, modified intent-to-treat; PRA, panel reactive antibody; PR-Tac, prolonged release tacrolimus; SD, standard deviation.

### Efficacy—Tacrolimus Dosage

Mean (SD) tacrolimus TDD, trough levels and trough:TDD are presented for the LCPT group and IR-Tac/PR-Tac groups, as well as for each tacrolimus formulation separately (Table 2).

**TABLE 2:** Tacrolimus TDD, trough levels and trough:TDD by period (mITT population).

TDD, mean (SD), mg	LCPT (N = 200)	IR-Tac/PR-Tac (N = 201)	IR-Tac (N = 86)	PR-Tac (N = 115)
Week 3 to Month 6	5.17 (2.97)	6.28 (3.56)	5.54 (2.91)	6.81 (3.88)
Overall	5.85 (3.08)	6.96 (3.65)	6.33 (3.24)	7.43 (3.88)
Week 1	10.96 (3.08)	11.72 (3.16)	11.34 (3.02)	12.01 (3.26)
Week 2	8.75 (4.01)	9.54 (4.55)	8.76 (3.94)	10.10 (4.87)
Week 3	8.07 (4.20)	9.20 (4.86)	8.17 (3.78)	9.93 (5.40)
Week 4	7.41 (4.02)	8.57 (4.64)	7.47 (3.66)	9.36 (5.11)
Months 1–3	5.80 (3.27)	7.00 (3.80)	6.23 (3.37)	7.56 (4.01)
Months 3–6	4.45 (2.87)	5.44 (3.23)	4.82 (2.74)	5.91 (3.50)
**Trough Levels, Mean (SD), ng/ml**				
Week 3 to Month 6	9.40 (1.72)	9.00 (1.67)	8.86 (1.51)	9.11 (1.78)
Overall	10.69 (2.58)	10.11 (2.12)	10.60 (2.46)	9.76 (1.76)
Week 1	13.96 (5.91)	13.07 (5.05)	14.59 (5.22)	11.94 (4.63)
Week 2	10.65 (3.67)	9.66 (3.60)	10.24 (3.54)	9.24 (3.60)
Week 3	10.70 (4.42)	9.91 (3.38)	10.45 (3.11)	9.52 (3.52)
Week 4	10.47 (3.52)	9.96 (3.04)	9.76 (2.54)	10.12 (3.38)
Months 1–3	9.69 (2.22)	9.36 (2.42)	9.23 (2.70)	9.45 (2.27)
Months 3–6	8.37 (1.87)	8.04 (1.78)	7.84 (1.89)	8.21 (1.69)
**Trough:TDD Mean (SD), ng/ml mg^−1^**				
Week 3 to Month 6	2.26 (1.38)	1.69 (0.85)	1.90 (0.97)	1.54 (0.73)
Week 1	1.22 (0.69)	1.09 (0.63)	1.29 (0.69)	0.94 (0.54)
Week 2	1.46 (0.99)	1.26 (0.91)	1.38 (0.70)	1.18 (1.03)
Week 3	1.68 (1.14)	1.33 (0.86)	1.58 (1.07)	1.16 (0.62)
Week 4	1.77 (1.08)	1.44 (0.93)	1.66 (1.13)	1.27 (0.69)
Months 1–3	2.23 (1.51)	1.68 (0.96)	1.91 (1.09)	1.51 (0.82)
Months 3–6	2.62 (1.80)	1.87 (0.95)	2.06 (1.05)	1.72 (0.83)

IR-Tac, immediate release tacrolimus; LCPT, LCP tacrolimus; mITT, modified intent-to-treat; PR-Tac, prolonged release tacrolimus; SD, standard deviation; TDD, total daily dose.

#### TDD

The mean (SD) tacrolimus TDD from Week 3 to Month 6 after transplant (primary endpoint) was lower in the LCPT group than in the IR-Tac/PR-Tac group: 5.17 (2.97) mg versus 6.28 (3.56) mg, respectively [IR-Tac: 5.54 (2.91) mg; PR-Tac: 6.81 (3.88) mg] (Table 2). The LS mean tacrolimus TDD from Week 3 to Month 6 after transplant was significantly lower in the LCPT group (5.14 mg) than in the IR-Tac/PR-Tac group (6.24 mg): −1.11 (LS mean difference, LCPT-IR-Tac/PR-Tac), −1.76, −0.45 (95% CI) (*p* < 0.001, Table 3).

**TABLE 3:** Tacrolimus TDD (mITT).

TDD	LCPT	IR-Tac/PR-Tac	Difference (LCPT—IR-Tac/PR-Tac)	
	(N = 200)	(N = 201)	LS mean (95% CI)	*p*-value
Week 3 to Month 6 (primary endpoint)				
LS mean, mg^a^	5.14	6.24	−1.11 (−1.76, −0.45)	<0.001
Whole study period				
LS mean, mg				
Overall^a^	5.82	6.92	−1.11 (−1.77, −0.45)	0.001
Week 1^b^	10.91	11.67	−0.75 (−1.35, −0.16)	0.013
Week 2^b^	8.71	9.50	−0.79 (−1.62, 0.05)	0.064
Week 3^b^	8.04	9.12	−1.08 (−1.98, −0.19)	0.018
Week 4^b^	7.35	8.52	−1.18 (−2.05, −0.31)	0.008
Months 1−3^b^	5.71	6.91	−1.20 (−1.91, −0.49)	0.001
Months 3−6^b^	4.39	5.37	−0.98 (−1.60, −0.36)	0.002
Week 3 to Month 6 normalized for weight				
Mean (SD), mg/kg	0.07 (0.04)	0.09 (0.05)		

a ANOVA model including treatment and country as fixed effects.

b MMRM model including treatment, period, treatment by period interaction, and country as fixed effects.

Week 3 to Month 6: mean calculation normalized for weight, n = 186 (LCPT) and 187 (IR-Tac/PR-Tac).

Whole study period: mean calculation, n = 200 (LCPT) and 201 (IR-Tac/PR-Tac); LS mean calculation, n = 401 (overall), 401 (Week 1), 391 (Week 2), 388 (Week 3), 384 (Week 4), 378 (Months 1–3), and 365 (Months 3–6).

ANOVA, analysis of variance; CI, confidence interval; IR-Tac, immediate release tacrolimus; LCPT, LCP tacrolimus; LS, least squares; mITT, modified intent-to-treat; MMRM, mixed model for repeated measures; PR-Tac, prolonged release tacrolimus; SD, standard deviation; TDD, total daily dose.

**FIGURE 2:**
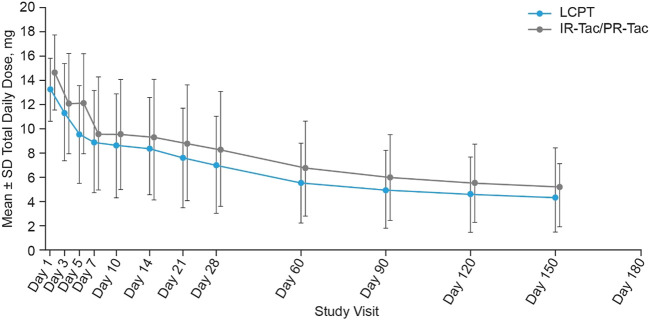
Tacrolimus total daily dose at each study visit (mean ± SD, mITT). Mean daily dose data was not collected at Day 180. IR-Tac, immediate release tacrolimus; LCPT, LCP tacrolimus; mITT, modified intent-to-treat; PR-Tac, prolonged release tacrolimus; SD, standard deviation.

Similar results were observed across the whole study period, overall and at each study visit (Table 2; Figure 2). At each time period, except Week 2, the LS mean TDD was significantly lower in the LCPT group than the IR-Tac/PR-Tac group (Table 3). Mean TDD normalized for weight was lower in the LCPT group than the IR-Tac/PR-Tac group (Table 3).

#### Trough Levels

**FIGURE 3:**
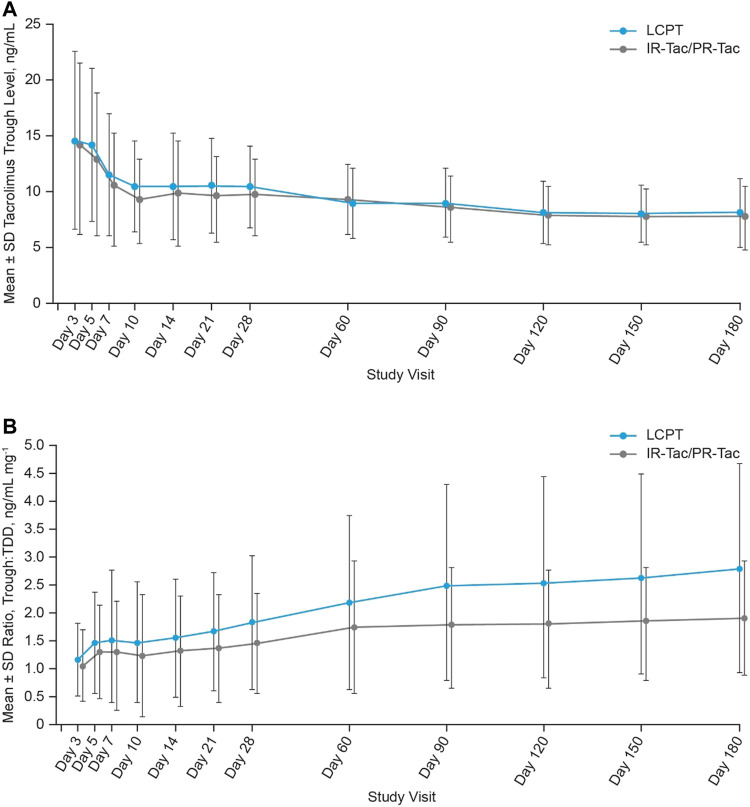
Tacrolimus trough levels **(A)** and trough:TDD **(B)** at each study visit (mean ± SD, mITT). IR-Tac, immediate release tacrolimus; LCPT, LCP tacrolimus; mITT, modified intent-to-treat; PR-Tac, prolonged release tacrolimus; SD, standard deviation; TDD, total daily dose.

Mean tacrolimus trough levels were higher in the LCPT group compared with the IR-Tac/PR-Tac group at each visit, except for Day 60 (Table 2; Figure 3A). LS mean tacrolimus trough levels were significantly higher in the LCPT group than the IR-Tac/PR-Tac group from Week 3 to Month 6: 0.41 (LS mean difference, LCPT-IR-Tac/PR-Tac), 0.08, 0.74 (95% CI) (*p* = 0.016, Table 4), and overall 0.62 (LS mean difference, LCPT-IR-Tac/PR-Tac), 0.17, 1.06 (95% CI) (*p* = 0.007, Table 4). The proportion of patients with trough levels within the standard reference range (5–15 ng/ml within the first 3 months after transplantation and 5–10 ng/ml thereafter) rose at each study visit from approximately 50% at Day 3 to >80% by Day 10. The proportion of trough level assessments within the standard range was similar in the LCPT and IR-Tac/PR-Tac groups (74.1 and 77.9%, respectively).

**TABLE 4:** Tacrolimus trough levels (mITT).

	No. patients	LCPT (N = 200)	IR-Tac/PR-Tac (N = 201)	Difference (LCPT—IR-Tac/PR-Tac)	
		LS mean, ng/ml	LS mean, ng/ml	LS mean (95% CI), ng/ml	*p*-value
Week 3 to Month 6	385	9.43	9.02	0.41 (0.08, 0.74)	0.016
Overall^a^	398	10.73	10.12	0.62 (0.17, 1.06)	0.007
Week 1^b^	397	13.99	13.06	0.93 (−0.14, 2.00)	0.090
Week 2^b^	389	10.68	9.68	1.01 (0.29, 1.72)	0.006
Week 3^b^	352	10.76	9.95	0.81 (−0.00, 1.61)	0.050
Week 4^b^	334	10.51	9.91	0.60 (−0.09, 1.29)	0.090
Months 1–3^b^	376	9.71	9.36	0.36 (−0.11, 0.82)	0.132
Months 3–6^b^	364	8.34	8.04	0.30 (−0.07, 0.67)	0.112

a ANOVA model including treatment and country as fixed effects.

b MMRM model including treatment, period, treatment by period interaction, and country as fixed effects.

ANOVA, analysis of variance; CI, confidence interval; IR-Tac, immediate release tacrolimus; LCPT, LCP tacrolimus; LS, least squares; mITT, modified intent-to-treat; MMRM, mixed model for repeated measures; PR-Tac, prolonged release tacrolimus.

The LS mean ratios of tacrolimus trough:TDD were significantly higher in the LCPT group than the IR-Tac/PR-Tac group at each study visit and during each period [Table 5; mean (SD) data is shown in Figure 3B].

**TABLE 5:** Tacrolimus trough:TDD (mITT).

	No. patients	LCPT (N = 200)	IR-Tac/PR-Tac (N = 201)	Difference (LCPT—IR-Tac/PR-Tac)	
		LS mean, ng/ml mg^−1^	LS mean, ng/ml mg^−1^	LS mean (95% CI), ng/ml mg^−1^	*p*-value
Week 3 to Month 6^a^	385	2.27	1.70	0.57 (0.34, 0.80)	<0.001
Week 1^b^	396	1.22	1.09	0.14 (0.01, 0.26)	0.034
Week 2^b^	389	1.47	1.26	0.21 (0.02, 0.40)	0.030
Week 3^b^	352	1.67	1.32	0.34 (0.14, 0.54)	<0.001
Week 4^b^	334	1.82	1.46	0.36 (0.15, 0.57)	<0.001
Months 1–3^b^	376	2.27	1.70	0.57 (0.31, 0.82)	<0.001
Months 3–6^b^	364	2.65	1.89	0.76 (0.47, 1.06)	<0.001

a ANOVA model including treatment and country as fixed effects.

b MMRM model including treatment, period, treatment by period interaction, and country as fixed effects.

ANOVA, analysis of variance; CI, confidence interval; IR-Tac, immediate release tacrolimus; LCPT, LCP tacrolimus; LS, least squares; mITT, modified intent-to-treat; MMRM, mixed model for repeated measures; PR-Tac, prolonged release tacrolimus; TDD, total daily dose.

#### Dose Adjustments

With the exception of 2 subjects each in both the LCPT and IR-Tac/PR-Tac groups, all subjects had dose adjustments. For all time periods, the mean number of dose adjustments was <3 for patients in both the LCPT and IR-Tac/PR-Tac groups, with no notable differences between treatment groups at each period.

#### Exploratory Dosage Endpoints

Compared with IR-Tac, a similar dose of LCPT resulted in statistically higher tacrolimus trough levels. The LS mean tacrolimus TDD from Week 3 to Month 6 after transplant was similar: 5.19 and 5.28 mg respectively for LCPT and IR-Tac; 0.092 (LS mean difference, LCPT-IR-Tac), -0.91, 0.73 (95% CI) (*p* = 0.825, Table 6 and Supplementary Figure S1A). LS mean tacrolimus trough levels were significantly higher with LCPT than IR-Tac from Week 3 to Month 6: 9.4 and 8.9 ng/ml respectively for LCPT and IR-Tac; 0.50 (LS mean difference, LCPT-IR-Tac), 0.05, 0.95 (95% CI) (*p* = 0.030, Table 6 and Supplementary Figure S1A). The LS mean ratios of tacrolimus trough:TDD were numerically, but not statistically, higher with LCPT compared with IR-Tac from Week 3 to Month 6: 2.25 vs 2.0 ng/ml mg^−1^ respectively for LCPT and IR-Tac, 0.25 (LS mean difference, LCPT-IR-Tac), −0.11, 0.60 (95% CI) (*p* = 0.172, Table 6 and Supplementary Figure S1A).

**TABLE 6:** Exploratory dosage endpoints: LCPT vs. IR-Tac (mITT).

Exploratory endpoints	LCPT	IR-Tac	Difference (LCPT—IR-Tac)	
Week 3 to Month 6	(N = 200)	(N = 86)	LS mean (95% CI)	*p*-value
Tacrolimus TDD				
LS mean, mg^a^	5.19	5.28	−0.09 (−0.91, 0.73)	0.825
Tacrolimus trough levels				
LS mean, ng/ml	9.4	8.9	0.50 (0.05, 0.95)	0.030
Ratio of tacrolimus trough level over TDD				
LS mean, ng/ml mg^−1^	2.25	2.00	0.25 (−0.11, 0.60)	0.172

a ANOVA model including treatment and country as fixed effects. Difference in LS means calculated by [(LCPT)–(IR-Tac or PR-Tac)].

ANOVA, analysis of variance; CI, confidence interval; IR-Tac, immediate release tacrolimus; LCPT, LCP tacrolimus; LS, least squares; mITT, modified intent-to-treat; MMRM; PR-Tac, prolonged release tacrolimus; TDD, total daily dose.

Compared with PR-Tac, a significantly lower dose of LCPT was required to achieve similar tacrolimus trough levels. LS mean tacrolimus TDD from Week 3 to Month 6 after transplant was significantly lower with LCPT: 5.15 and 7.04 mg respectively for LCPT and PR-Tac; −1.89 (LS mean difference, LCPT-PR-Tac), −2.68, −1.10 (95% CI) (*p* < 0.001, Table 7 and Supplementary Figure S1B). LS mean tacrolimus trough levels were similar from Week 3 to Month 6: 9.4 and 9.2 ng/ml respectively for LCPT and PR-Tac; 0.21 (LS mean difference, LCPT-PR-Tac), −0.19, 0.62 (95% CI) (*p* = 0.298, Table 7 and Supplementary Figure S1B). The LS mean ratios of tacrolimus trough:TDD were significantly higher with LCPT compared with PR-Tac from Week 3 to Month 6: 2.26 vs 1.49 ng/ml mg^−1^ respectively for LCPT and PR-Tac, 0.78 (LS mean difference, LCPT-PR-Tac), 0.50, 1.06 (95% CI) (*p* < 0.001, Table 7 and Supplementary Figure S1B).

**TABLE 7:** Exploratory dosage endpoints: LCPT vs PR-Tac (mITT).

Exploratory endpoints	LCPT	PR-Tac	Difference (LCPT—PR-Tac)	
Week 3 to Month 6	(N = 200)	(N = 115)	LS mean (95% CI)	*p*-value
Tacrolimus TDD				
LS mean, mg^a^	5.15	7.04	−1.89 (−2.68, −1.10)	<0.001
Tacrolimus trough levels				
LS mean, ng/ml	9.4	9.2	0.21 (−0.19, 0.62)	0.298
Ratio of tacrolimus trough level over TDD				
LS mean, ng/ml mg^−1^	2.26	1.49	0.78 (0.5, 1.06)	<0.001

a ANOVA model including treatment and country as fixed effects. Difference in LS means calculated by [(LCPT)−(IR-Tac or PR-Tac)].

ANOVA, analysis of variance; CI, confidence interval; IR-Tac, immediate release tacrolimus; LCPT, LCP tacrolimus; LS, least squares; mITT, modified intent-to-treat; MMRM; PR-Tac, prolonged release tacrolimus; TDD, total daily dose.

### Efficacy—Clinical Outcomes

There were no statistically significant differences between the LCPT and IR-Tac/PR-Tac groups overall or in any measure of treatment failure (death, graft failure, biopsy-proven acute rejection, or loss to follow-up; Table 8). Eighteen patients in each group (9.0%) experienced treatment failure, mainly biopsy-proven acute rejection [occurring in 12 (6.0%) patients in the LCPT group and 10 (5.0%) in the IR-Tac/PR-Tac group]. There were no statistically significant differences between the LCPT and IR-Tac/PR-Tac groups in time to treatment failure or time to treatment discontinuation (log-rank *p* = 0.965 and *p* = 0.461, respectively). Overall, the number (%) of subjects with treatment failure was 18 (9.0%) for LCPT, 7 (8.1%) for IR-Tac and 11 (9.6%) for PR-Tac; no significant difference was detected between the LCPT and IR-Tac subgroup (estimate 0.9; 95% CI: −7.5, 7.2; *p*-value: >0.999) or between the LCPT and PR-Tac subgroup (estimate −0.6; 95% CI: −8.1, 5.8; *p*-value: 0.843; Supplementary Tables S1A,B).

**TABLE 8:** Patients with treatment failure (mITT).

	LCPT (N = 200)	IR-Tac/PR-Tac (N = 201)	Difference (LCPT—IR-Tac/PR-Tac)	
	n (%)	n (%)	Estimate (95% CI), %	*p*-value
Overall treatment failure	18 (9.0)	18 (9.0)	0.0 (−5.7, 5.8)	>0.999
Death	4 (2.0)	4 (2.0)	0.0 (−3.2, 3.3)	>0.999
Graft failure	4 (2.0)	4 (2.0)	0.0 (−3.2, 3.3)	>0.999
Biopsy-proven acute rejection	12 (6.0)	10 (5.0)	1.0 (−3.7, 5.8)	0.668
Loss to follow-up	0	0	NE	NE

Two patients in the LCPT group experienced two events each (graft failure and biopsy-proven acute rejection).

*p*-value based on 2-sided Fisher’s exact test; 95% CI based on the Newcombe-Wilson method.

CI, confidence interval; IR-Tac, immediate release tacrolimus; LCPT, LCP tacrolimus; mITT, modified intent-to-treat; NE, not estimable; PR-Tac, prolonged release tacrolimus.

**FIGURE 4:**
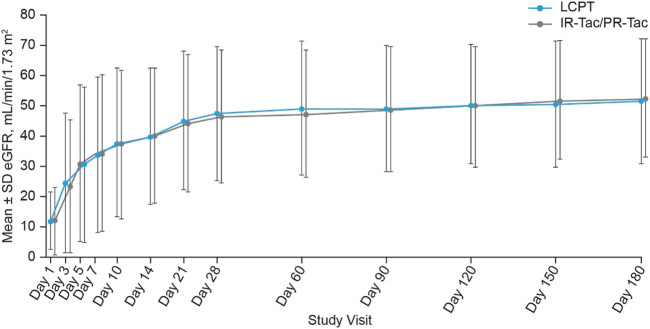
Estimated glomerular filtration rate at each study visit (mean ± SD, mITT). eGFR, estimated glomerular filtration rate; IR-Tac, immediate release tacrolimus; LCPT, LCP tacrolimus; mITT, modified intent-to-treat; PR-Tac, prolonged release tacrolimus; SD, standard deviation.

There were no statistically significant differences observed between the LCPT and IR-Tac/PR-Tac groups in the number of patients who experienced delayed graft function [23 (11.5%) and 22 (10.9%), respectively, *p* = 0.876] or the number of patients with rejection assessed as acute by the investigator [7 (3.5%) and 6 (3.0%), respectively, *p* = 0.787]. Biopsy-proven acute rejection was the reason for treatment failure in 12 (6.0%) patients in the LCPT group and 10 (5.0%) patients in the IR-Tac/PR-Tac group. In addition, no statistically significant differences in estimated glomerular filtration rates (eGFR) were shown between LCPT and IR-Tac/PR-Tac treatment groups at any post-baseline visit (Figure 4). The number (%) of subjects with delayed graft function was 23 (11.5%) for LCPT, 4 (4.7%) for IR-Tac and 18 (15.7%) for PR-Tac. No significant difference was detected between the LCPT and IR-Tac subgroups (estimate 6.8; 95% CI: −0.8, 12.7; *p*-value: 0.079) or between the LCPT and PR-Tac subgroups (estimate −4.2; 95% CI: −12.7, 3.4; *p*-value: 0.301) (Supplementary Tables S1A,B). The number (%) of subjects with local diagnosis of acute rejection requiring treatment was 7 (3.5%) for LCPT, 2 (2.3%) for IR-Tac and 4 (3.5%) for PR-Tac. No significant difference was detected between the LCPT and IR-Tac subgroup (estimate 1.2; 95% CI: −4.9, 5.1; *p*-value: 0.729) or between the LCPT and PR-Tac subgroup (estimate 0.0; 95% CI: −5.4, 4.2; *p*-value: >0.999) (Supplementary Tables S1A,B).

The most common concomitant immunosuppressants were glucocorticoids [taken by 193 (96.5%) and 194 (96.5%) patients in the LCPT and IR-Tac/PR-Tac groups, respectively], and basiliximab [117 (58.5%) and 121 (60.2%) patients; Table 9]. Mycophenolate, either mofetil or sodium, was used by 167 (83.5%) and 175 (87.1%) patients in the LCPT and IR-Tac/PR-Tac groups, respectively.

**TABLE 9:** Concomitant immunosuppressant medications (mITT).

Subjects, n (%)	LCPT (N = 200)	IR-Tac/PR-Tac (N = 201)
Glucocorticoids and corticosteroid NOS	193 (96.5)	194 (96.5)
Immunosuppressants	155 (77.5)	166 (82.6)
Antithymocyte immunoglobulin	1 (0.5)	1 (0.5)
Belatacept	1 (0.5)	1 (0.5)
Everolimus	4 (2.0)	2 (1.0)
Mycophenolate mofetil and sodium	167 (83.5)	175 (87.1)
Basiliximab	117 (58.5)	121 (60.2)
Ciclosporin	1 (0.5)	1 (0.5)
Azathioprine	0	2 (1.0)

Subjects may have more than one medication. Concomitant medications were coded with the WHO Drug dictionary dated December 2014.

IR-Tac, immediate release tacrolimus; LCPT, LCP tacrolimus; mITT, modified intent-to-treat; NOS, not otherwise specified; PR-Tac, prolonged release tacrolimus; WHO, World Health Organization.

### Safety

The safety profile of LCPT was similar to that of IR-Tac/PR-Tac and to that of the two formulations separately, and no new unexpected safety warnings were observed (Table 10). The most commonly reported AEs considered possibly related to treatment were tremor (13.5 and 9.0% in the LCPT and IR-Tac/PR-Tac groups, respectively), cytomegalovirus infection (4.5 and 3.5%), urinary tract infection (3.0 and 2.5%), and post-transplant diabetes mellitus (2.0 and 4.0%, defined as the need for any antidiabetic agent and/or HbA_1c_ >6.5% at Months 3 and 6). BK virus infections occurred in 11 (5.5%) and 12 (6.0%) of patients in the LCPT and IR-Tac/PR-Tac groups, respectively. A total of 99 patients (49.5%) in the LCPT group and 93 (46.3%) in the IR-Tac/PR-Tac groups experienced a serious adverse event (SAE). In the LCPT group, the most common SAEs were complications of the transplanted kidney (6.0%), raised blood creatinine (5.0%), transplant rejection (4.5%), and urinary tract infection (3.0%). In the IR-Tac/PR-Tac group, the most common SAEs were urinary tract infection (5.0%), transplant rejection (4.0%), and diarrhea (3.5%). Four (2%) patients in each study group died. Events leading to death in the LCPT group were duodenal ulcer, pancreatitis and sepsis (in one patient), intestinal ischemia, sequelae of a complicated mycotic aneurysm of the graft artery, and multi-organ failure. Events leading to death in the IR-Tac/PR-Tac group were acute respiratory distress syndrome, cardiac arrest, multi-organ failure, and myocardial infarction. There were no notable differences in the effects of LCPT and IR-Tac/PR-Tac on vital signs, ECG, or clinical laboratory results, including lipid profiles and blood pressure parameters.

**TABLE 10:** Treatment-emergent adverse events (TEAE) in the safety population.

Subjects (%) [E]	LCPT (N = 200)	IR-Tac/PR-Tac (N = 201)	IR-Tac (N = 86)	PR-Tac (N = 115)
Any TEAE	195 (97.5) [1704]	192 (95.5) [1546]	82 (95.3) [637]	110 (95.7) [909]
Any treatment-emergent ADR	73 (36.5) [164]	77 (38.3) [141]	43 (50.0) [86]	34 (29.6) [55]
Any serious TEAE	99 (49.5) [185]	93 (46.3) [178]	40 (46.5) [68]	53 (46.1) [110]
Any serious TEADR	26 (13.0) [34]	23 (11.4) [28]	13 (15.1) [18]	10 (8.7) [10]
Any severe TEAE	48 (24.0) [92]	59 (29.4) [97]	29 (33.7) [46]	30 (26.1) [51]
Any TEAE leading to discontinuation	12 (6.0) [15]	16 (8.0) [16]	8 (9.3) [8]	8 (7.0) [8]
Any treatment-emergent ADR leading to discontinuation	3 (1.5) [3]	4 (2.0) [4]	2 (2.3) [2]	2 (1.7) [2]
Any AE leading to death	4 (2.0) [6]	4 (2.0) [4]	1 (1.2) [1]	3 (2.6) [3]

E, number of events; ADR, adverse drug reaction; AE, adverse event; IR-Tac, immediate release tacrolimus; LCPT, LCP tacrolimus; PR-Tac, prolonged release tacrolimus; TEAE, treatment emergent AE.

## Discussion

This is the first study comparing LCPT versus tacrolimus standard-of-care in *de novo* kidney transplant recipients in real-life clinical practice across Europe. The results showed that LCPT can achieve similar clinical outcomes to other tacrolimus formulations, with a lower daily dose. The study met its primary objective by demonstrating a significantly lower mean tacrolimus TDD with LCPT than with IR-Tac/PR-Tac from Week 3 to Month 6. The 6-months timeframe for this study was chosen to be in line with similar studies assessing biopsy-proven acute rejection following transplantation, and with the assumption that it would take 3 weeks to stabilize tacrolimus dose levels post-transplantation (22–24).

TDD was significantly lower with LCPT than with IR-Tac/PR-Tac throughout the study period, and when normalized for weight. Despite the lower dose required, patients receiving LCPT maintained significantly higher tacrolimus trough levels than those receiving standard-of-care while importantly remaining within the standard reference range, leading to a higher ratio of tacrolimus trough:TDD in the LCPT group.

For all other secondary efficacy endpoints, there were no notable differences between the two treatment groups. The overall number of treatment failures and rejections was low; approximately 9% of patients in each treatment group experienced treatment failure (a composite of death, graft failure, biopsy-proven acute rejection, or loss to follow-up), approximately 6% had biopsy-proven acute rejection, and approximately 11% experienced delayed graft function. These results are in line with the low treatment failure rates seen in *de novo* kidney recipients receiving LCPT or IR-Tac in a 12-months study (14).

The safety profiles of LCPT and tacrolimus standard-of-care were similar, and no new unexpected safety warnings were observed. The most common treatment-related AEs in both treatment groups were tremor, cytomegalovirus infection, urinary tract infection, and diabetes mellitus.

Previous studies have also reported a lower TDD with LCPT compared with IR-Tac or PR-Tac (14, 17, 20, 21), in addition to lower rates of efficacy failure among high-risk subgroups, including black recipients and recipients ≥65 years of age (25). Non-inferiority of LCPT versus IR-Tac with respect to treatment failure has been previously shown in stable kidney transplant patients who converted from IR-Tac to LCPT (20). Non-inferiority of LCPT in *de novo* transplant patients has also been demonstrated at 1 year after transplantation (14) with similar efficacy and safety maintained over 2 years (21). The present study extends the existing knowledge to include comparison with PR-Tac in *de novo* patients, demonstrating that LCPT has similar efficacy to both IR-Tac and PR-Tac in this population.

The lower dose and higher trough levels observed with LCPT in the present study may be attributed to improved bioavailability resulting from controlled release of tacrolimus. This study did not assess bioavailability directly, however previous studies have demonstrated significantly higher bioavailability and lower peak-to-trough fluctuation with LCPT compared with PR-Tac (18). Lower tacrolimus bioavailability has been reported in women and African Americans, largely due to variations in hepatic CYP3A4 content and *CYP3A5* gene expression (26–29). It has also been suggested that elderly transplant recipients may have greater variability in tacrolimus levels compared with younger patients (30); therefore, elderly patients may particularly benefit from the improved pharmacokinetic profile of LCPT, as previously indicated by a subgroup analysis (25).

Given the different immunosuppressive regimens available, there is a need to increase the use of support systems and biomarkers to help improve clinical decision making and to monitor outcomes. Although recent pharmacokinetic studies have highlighted the major influence of CYP3A genotype on tacrolimus exposure (31–33), CYP3A phenotype did not explain all pharmacokinetic variability, perhaps because multiple factors drive inter-individual variability in tacrolimus metabolism (11, 31–33). Continued investigation of optimal management algorithms is needed, and accordingly, a potential tool to assess risk factors for poor long-term outcomes has been proposed based on the concept of individual metabolic rates. This tool showed that fast tacrolimus metabolism, defined as having a low ratio of tacrolimus trough:TDD, associates with reduced survival rates of patients, lower renal function, and infection, suggesting that some patients may benefit from alternative immunosuppressive regimens or concepts (34–36).

Once-daily dosing may represent a further advantage of LCPT and PR-Tac over IR-Tac. Transplant recipients are often reported to be non-adherent to immunosuppressive therapy (37, 38), and once-daily tacrolimus has been shown to be associated with improved adherence (39, 40). This is key for successful treatment outcomes, particularly for therapies such as tacrolimus that have a narrow therapeutic window. Improvements in adherence with once-daily dosing could not be evaluated in the present study, because the tacrolimus standard-of-care control arm allowed use of both twice-daily IR-Tac (86 patients) and once-daily PR-Tac (115 patients). The prespecified subgroup analysis confirmed that LCPT has a clinically relevant greater bioavailability compared to the other oral formulations of tacrolimus, and that this difference in bioavailability of LCPT is particularly significant in comparison with PR-Tac.

A key strength of the study is that it reflected real-life conditions across a number of different countries for *de novo* kidney transplant patients, in that investigators were free to choose IR-Tac or PR-Tac for the comparator arm according to their usual clinical practice. The results therefore provide a representative picture of the potential benefits of LCPT compared with tacrolimus standard-of-care as routinely implemented in transplant centers across Europe. A limitation is that the study included mainly white, middle-aged men with standard immunological risk for graft rejection, and the results may not be generalizable to the overall kidney transplant population.

In conclusion, the study demonstrated that LCPT, when administered to *de novo* kidney transplant patients, allows a lower TDD than current standard-of-care tacrolimus, while maintaining gold-standard levels of clinical outcomes.

## Data Availability

The datasets presented in this article are not readily available. Chiesi access criteria and complete process for clinical data sharing is available on the Chiesi Group website. Requests to access the datasets should be directed to https://www.chiesi.com/en/chiesi-clinical-trial-data-request-portal/.

## Abbreviations

AE, adverse event; ANOVA, analysis of variance; CI, confidence interval; ECG, electrocardiogram; eGFR, estimated glomerular filtration rate; HLA, human leukocyte antigen; IR-Tac, intermediate-release tacrolimus; LCPT, LCP-tacrolimus; LS, least squares; MMRM, mixed model for repeated measures; mITT, modified intent-to-treat; NE, not estimable; PR-Tac, prolonged-release tacrolimus; PRA, panel reactive antibody; SAE, serious adverse event; SD, standard deviation; STRATO, switching STudy of kidney tRansplant pAtients with Tremor to LCP-TacrO; TDD, total daily dose; TEAE, treatment-emergent adverse event.
