# Supplementary material for: Prolonged-Release Once-Daily Formulation of Tacrolimus Versus Standard-of-Care Tacrolimus in de novo Kidney Transplant Patients Across Europe
Source: Transpl Int. 2022 Mar 21;35:10225. doi: 10.3389/ti.2021.10225 (PMC9397503; doi:10.3389/ti.2021.10225)
Supplement: Supplementary file 1 [file Table1.docx]

**Supplementary Table 1A. Treatment failure: LCPT vs IR-Tac (mITT)**

|  | **LCPT (N=200)** | **IR-Tac**  **(N=85)** | **Difference (LCPT – IR-Tac)** | |
| --- | --- | --- | --- | --- |
|  | **n (%)** | **n (%)** | **Estimate (95% CI), %** | **p-value** |
| Overall treatment failure | 18 (9.0) | 7 (8.1) | 0.9 (-7.5, 7.2) | >0.999 |
| Delayed graft function | 23 (11.5) | 4 (4.7) | 6.8 (-0.8, 12.7) | 0.079 |
| Local diagnosis of acute rejection | 7 (3.5) | 2 (2.3) | 1.2 (-4.9, 5.1) | 0.729 |

p-value based on 2-sided Fisher’s exact test; 95% CI based on the Newcombe-Wilson method.

CI, confidence interval; IR-Tac, immediate release tacrolimus; LCPT, LCP tacrolimus; mITT, modified intent-to-treat.

**Supplementary Table 1B. Treatment failure: LCPT vs PR-Tac (mITT)**

|  | **LCPT (N=200)** | **PR-Tac**  **(N=85)** | **Difference (LCPT – PR-Tac)** | |
| --- | --- | --- | --- | --- |
|  | **n (%)** | **n (%)** | **Estimate (95% CI), %** | **p-value** |
| Overall treatment failure | 18 (9.0) | 11 (9.6) | -0.6 (-8.1, 5.8) | 0.843 |
| Delayed graft function | 23 (11.5) | 18 (15.7) | -4.2 (-12.7, 3.4) | 0.301 |
| Local diagnosis of acute rejection | 7 (3.5) | 4 (3.5) | 0.0 (-5.4, 4.2) | >0.999 |

p-value based on 2-sided Fisher’s exact test; 95% CI based on the Newcombe-Wilson method.

CI, confidence interval; LCPT, LCP tacrolimus; mITT, modified intent-to-treat; PR-Tac, prolonged release tacrolimus.
