# Supplementary material for: Prolonged-Release Once-Daily Formulation of Tacrolimus Versus Standard-of-Care Tacrolimus in de novo Kidney Transplant Patients Across Europe
Source: Transpl Int. 2022 Mar 21;35:10225. doi: 10.3389/ti.2021.10225 (PMC9397503; doi:10.3389/ti.2021.10225)
Supplement: Supplementary file 2 [file Image1.pdf]

Supplementary Figure 1. Exploratory dosage endpoints: Forest plots comparing LCPT vs IR-Tac (A) and vs PR-Tac (B) (mITT)

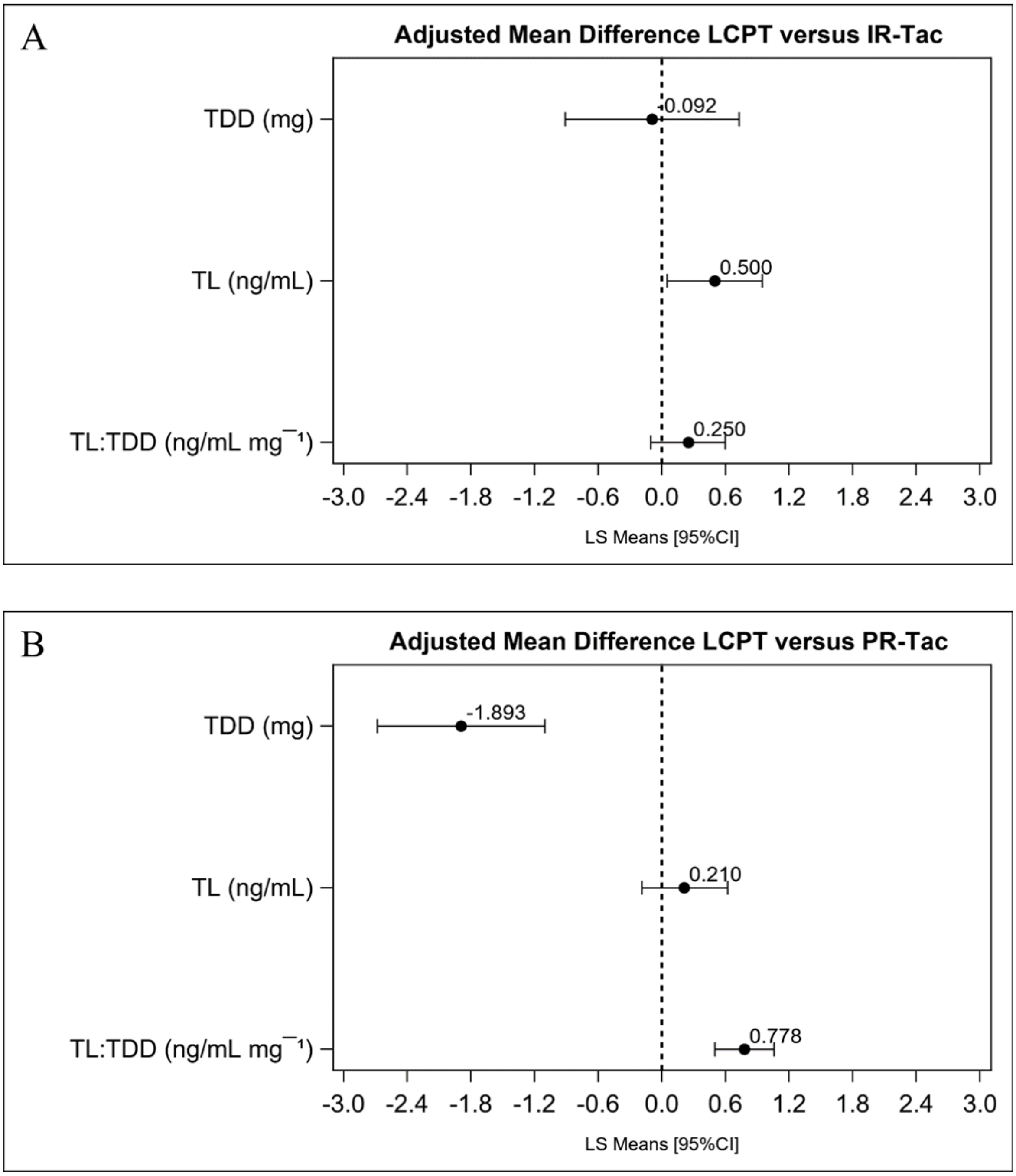

ANOVA model including treatment and country as fixed effects. Difference in LS means calculated by ([LCPT] – [IR-Tac or PR-Tac]).

ANOVA, analysis of variance; LCPT, LCP tacrolimus; IR-Tac, immediate release tacrolimus; PR-Tac, prolonged release tacrolimus; LS, least squares; mITT, modified intent-to-treat; TL, trough level; TDD, total daily dose.
